# An unusual presentation of ovarian carcinoma with supraclavicular lymph node and colorectal metastases leading to spontaneous rectovaginal fistula

**DOI:** 10.1016/j.ijscr.2023.108189

**Published:** 2023-04-13

**Authors:** Ikram Kharmach, Samia Malki, Ouissam Al Jarroudi, Tijani El Harroudi, Badr Serji, Said Afqir

**Affiliations:** a Department of Medical Oncology, Mohammed VI University Hospital, Oujda, Morocco; b Faculty of Medicine and Pharmacy, Mohammed I^st^ University, Oujda, Morocco; c Department of Anatomic Pathology, Mohammed VI University Hospital, Oujda, Morocco; d Department of Surgical Oncology, Mohammed VI University Hospital, Oujda, Morocco

**Keywords:** Metastatic ovarian cancer, Rectal metastasis, Rectovaginal fistula, Supraclavicular lymph node

## Abstract

**Introduction and importance:**

Secondary metastases to the rectum from primary ovarian cancer are a rare entity and their diagnosis and management are challenging. In this report, we discuss the findings of the case of metastatic ovarian cancer to supraclavicular lymph nodes and the rectum complicated with rectovaginal fistula.

**Case presentation:**

A 68-year-old woman was admitted for abdominal pain with rectal bleeding. Pelvic examination revealed a left latero-uterine mass. Abdominal-pelvic CT scan showed a tumor mass on the left ovary. A cytoreductive surgery and resection of a non-imaged rectal nodule identified during surgery were performed. The tumor specimens including the rectal metastasis were immunohistochemically confirming a metastatic ovarian cancer using CK7, WT1 and CK20. The patient received chemotherapy and had complete remission. However, she had a recto-vaginal fistula confirmed by imaging and had developed right supraclavicular lymphadenopathy from ovarian cancer later.

**Clinical discussion:**

The dissemination of ovarian cancer in the digestive tract can be frequently, through direct invasion, abdominal implantation and lymphatic system. Unusually, ovarian cancer cells may spread to supra-clavicular nodes, because of the connection of the two diaphragmatic stages allowing the lymph flows through the lymphatic vessels. Moreover, rectovaginal fistula is an uncommon complication which can be seen spontaneously or due to certain patient's features.

**Conclusion:**

In advanced ovarian carcinoma, it is required to properly assess the digestive tract during surgery because imaging can miss metastatic lesions such as our case. The use of immunohistochemistry is recommended to differentiate between primary ovarian carcinoma and secondary metastasis.

## Highlights

• Rectal metastasis from primary ovarian cancer is a rare condition.
• Immunohistochemistry helps differentiate primary and metastatic ovarian carcinoma.
• A proper assessment of the digestive tract is crucial to exclude synchronous lesions.
• Rectovaginal fistula is an uncommon complication in ovarian cancer.

## 1 Introduction

Ovarian cancer (OC) is one of the major causes of death among women diagnosed with reproductive tract malignancies and it mostly affects women after menopause and rarely before the age of 40 [1]. Patients with OC are often diagnosed at advanced stage particularly distant sites which is associated with high mortality rates [2]. The peritoneal cavity is the most frequent site for OC seeding. Although, other unusual and rare sites may be involved such as the Colo rectum and distant lymph nodes [2]. Several complications can be developed related to either treatment or surgery for OC such as rectovaginal fistula which can be seen spontaneously or associated with patient's features [3].

In this case report based on SCARE 2020 guidelines for better reporting [4], we discuss the findings of a patient who underwent surgery for an epithelial ovarian cancer and developed rectal metastasis and recto-vaginal fistula with particular attention to the impact of histopathological examination and periodic follow-up of patients with unusual metastatic sites.

## 2 Presentation of case

A 68-year-old Moroccan Amazigh housewife living in a rural area with normal body mass index presented hypogastric pain which was more accentuated on the left, associated with rectal bleeding of 2 weeks duration. The patient denied alcohol abuse or any history of smoking. She had also no previous social, gynecologic or family history of cancer. She took oral medication for diabetes (metformin at the dose of 1 g per day). She had a slim figure and her abdominal examination shows no abnormalities. On pelvic examination, there is a firm, irregular mass in the left latero-uterine area. There were no signs of perianal sepsis, or any palpable mass.

**Fig. 1:**
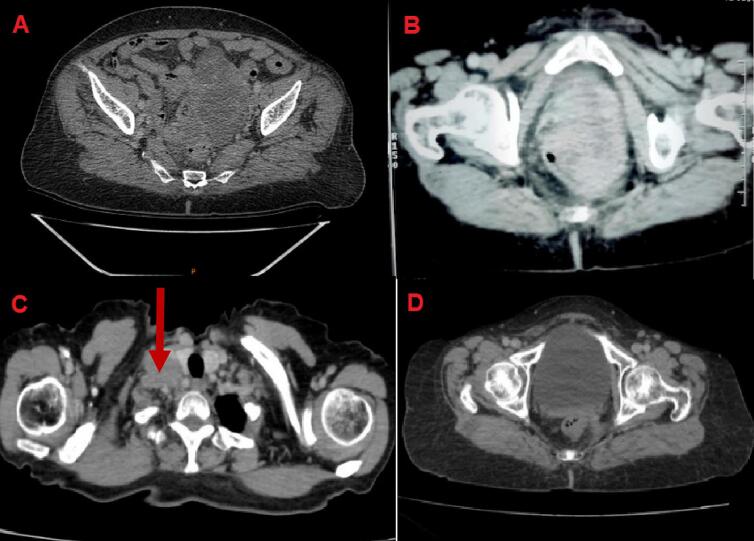
(A) CT scan image of the abdomen showed a left ovary mass, (B) CT scan image showing an irregular circumferential thickening of the rectum, (C) CT scan image of the thoracic-abdominal-pelvic level showing a right supra-clavicular adenopathy, and (D) CT scan image showing complete remission of the primary tumor.

**Fig. 2:**
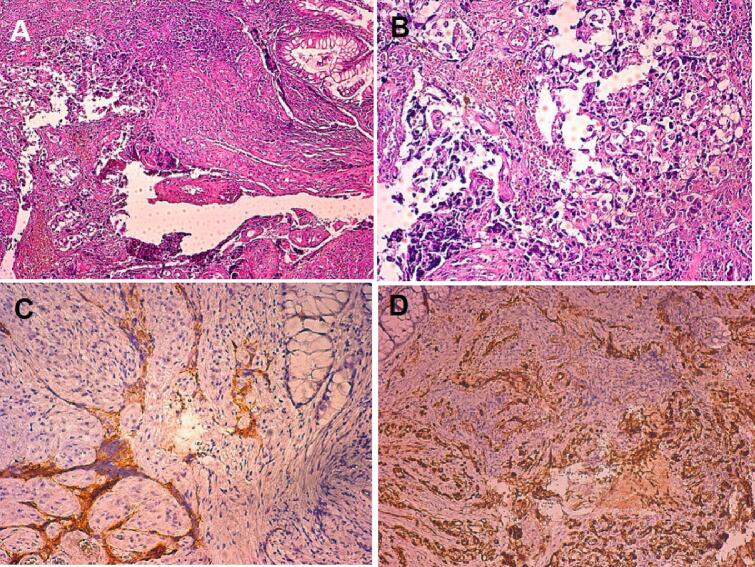
(A) microphotograph showing a colonic wall with carcinomatous proliferation in its submucosa arranged in clusters and micropapillary (HE, ×100), (B) tumor cells are atypical with hyperchromatic nuclei and abundant eosinophilic cytoplasm (HE, ×200), (C) the tumor cells express the anti-CK7 antibody and (D) tumor cells express anti-WT1 antibody.

A thoraco-abdominopelvic computed tomography (CT) scan showed a large heterogeneous polylobed mass measuring 80 mm suggesting a tumor of the left ovary, with no ascites or deep adenopathy (Fig. 1A). Cancer antigen 125 (CA-125) was prescribed but not done due to the low-income status of our patient. The patient was healthy enough to undergo anesthesia, no particular measures was taken before surgery. An exploratory laparotomy was performed under general anesthesia in the supine position by an experienced senior surgical oncologist surgeon. A left ovarian mass of malignant appearance, adherent to the sigmoid colon at the top of the wall and a nodule of peritoneal carcinomatosis invading the upper rectum were noticed. The surgical treatment included a total hysterectomy, a bilateral annexectomy, omentectomy and appendectomy involving a resection of the rectal nodule. Pelvic and lumbo-aortic lymph node dissection was not performed as per the latest recommendations. After debulking surgery, an abdominal-pelvic CT revealed an enlargement of the uterine cavity with a posterior invasion to the rectum (Fig. 1B). The initial anatomopathological study showed a tumor lesion measuring 8 × 3 × 2 cm, predominantly of solid cystic consistency (90 %). The cystic component was multilocular with the presence of endocytic vegetations. The capsule was intact with no invasion or exocytic vegetations. The patient underwent colonoscopy that revealed extended ulcerations of 1 to 9 cm from the anal margin. Immunohistochemistry (IHC) was performed and showed tumor cells expressing cytokeratin 7 (CK7) but negative for cytokeratin 20 (CK20), synaptophysin, progesterone receptors and cluster of differentiation 30 (CD30). The rectal resection was infiltrated with carcinoma (Fig. 2A and B).

The IHC analysis confirmed the ovarian origin of this infiltration and it was positive for CK7 and Wilms tumor 1 (WT1) and negative for CK20 (Fig. 2C and D). Based on these findings, high-grade serous adenocarcinoma of the ovary according to the world health organization classification (2020) was retained and classified as stage IVB according to the International Federation of Gynecology and Obstetrics (FIGO) staging system for ovarian cancer (8th edition,2017). Within a week, the patient was discharged from the hospital and continued treatment with antibiotics and preventive anticoagulation at home. The patient was lost to follow-up for 4 months and during her visit, a cervical thoraco-abdominopelvic CT scan was performed and revealed a right cervical tissue mass measuring 29 mm × 31 mm suggesting an adenopathy (Fig. 1C) which was confirmed on physical examination. A biopsy was performed and the histopathological examination of the supraclavicular nodule confirmed the secondary lymph node location of a known high-grade serous carcinoma in our patient. No homologous recombination deficiency testing was performed due to its unavailability at the hospital.

The patient received six cycles of combination chemotherapy consisting of paclitaxel at a dose of 175 mg/m^2^ and carboplatin AUC 6. Bevacizumab was not available and not affordable to our patient. The most common adverse event was neutropenia grade II with no fever managed successfully with treatment abstinence.

**Fig. 3:**
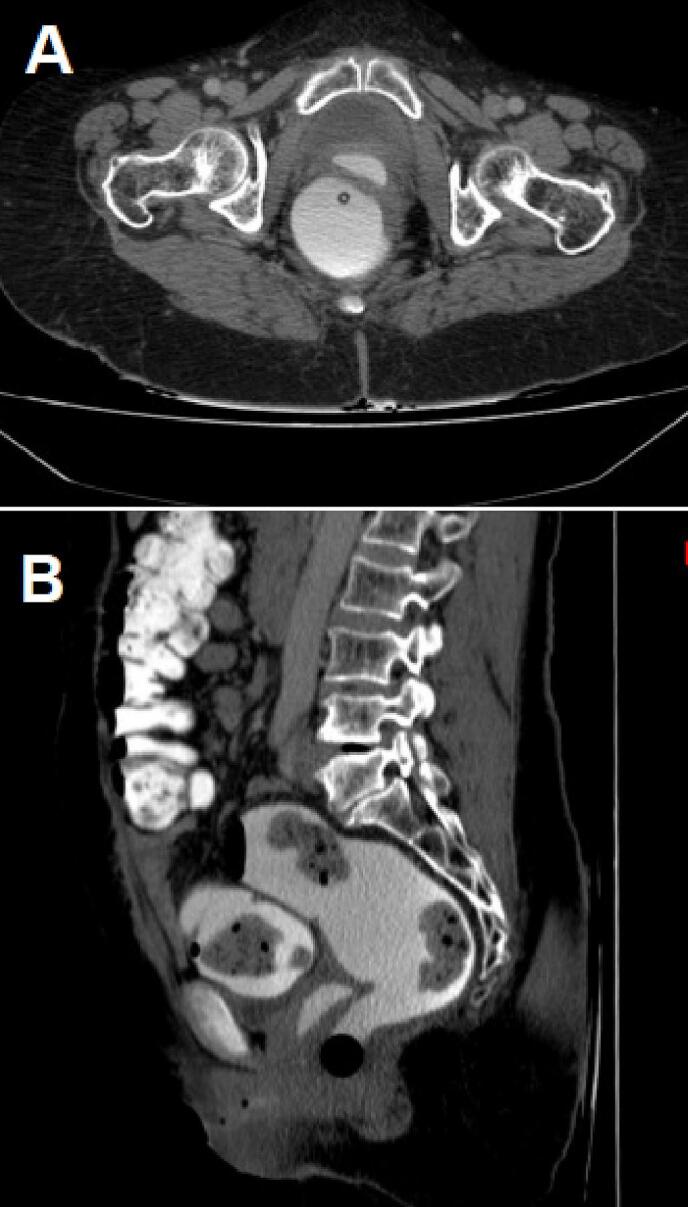
(A and B). CT scan images after opacification by vaginal route showing the passage of the contrast product at the vaginal level revealing a rectovaginal fistula.

Nine months after initial chemotherapy, the patient reported leakage of stool from the vagina. A CT scan revealed a rectovaginal fistula (Fig. 3) and almost complete remission of the primary tumor (Fig. 1D). An ostomy was proposed as a therapeutic option but rejected by the patient. She has been monitored regularly with clinical examination and CT scans every 3 months. No recurrent disease was detected at 15 months of follow up. Our patient is still alive at the time of writing this case report. On the other hand, our patient was satisfied with our management. However, she wished to have been treated with targeted agents such as bevacizumab and poly (ADP-ribose) polymerases (PARP) inhibitors which were not available in our under-resourced setting.

## 3 Discussion

OC accounts for approximately 5 % of all female cancers and it includes several histological types [1]. The majority of OCs are of epithelial origin. 90 % of cases fall into this category including mucinous, clear cell, endometrioid and serous carcinomas which represent the most common type [5]. In fact, unlike ovarian metastases of digestive origin such as the classic Krukenberg tumor characterized by the presence of signet ring cells in the ovaries which are more common [5]. According to Lindner et al. ovarian metastases account for about 7 % of all OCs and present a colorectal origin in about 45 % of cases [6]. To determine the etiological origin of the metastasis, not only the morphology of the tumor is required, but also the IHC study based on the expression of cytokeratins the tumor cells is essential because of the indistinguishable histological appearance of OCs from the primary gastrointestinal tumor.

In order to determine the cut-point between high- and low-grade OC, Manu et al. [7] showed in his study that tumor protein P53, P16 and WT1 had significantly higher staining scores in the group of ovarian serous carcinoma comparing to the benign group, furthermore, the association of moderate to high P53 and WT1 scores provides a solid argument to confirm high grade serous ovarian tumors. According to Chu et al. CK7+, CK20- represents 98 % of OCs versus 0 % of colorectal cancers. Beyond that, two markers were included in the study of Lagendijk et al. [8] who reached the same conclusions suggesting that CK7+, CA125+ /CK20-, carcino-embryonic antigen (CEA)- are biomarkers for diagnosing an ovarian origin, and CK7 - /CA-125 - /CK20 + /CEA+ in case of a colon origin. However, CA125 remains normal in 15 % of OCs. In our case, the combination of the morphological study and the results of the IHC analysis confirmed the primary ovarian origin.

The dissemination of OC in the digestive tract can take various routes frequently by a peritoneal route, which is the most common form. OC cells disseminate through the exfoliation of tumor cells in the cavity and are transported then by the ascitic fluid to the organs of the peritoneal cavity [9]. Subsequently, the invasion can occur directly by invading adjacent structures such as the colon. Then lymphatic route can also be involved, initially through the para-aortic nodes, followed by the broad ligament nodes which then drain into the hypogastric nodes. Finally, hematological dissemination, which is less frequent, usually occurs in the presence of advanced peritoneal disease and leads to the invasion of the pleura, the lung, the liver and rarely the kidneys [5].

The interval time between diagnosis of OC and identification of metastases is usually delayed, it ranged from 1 to 139 months and the survival time after diagnosis of dissemination ranged from 1 to 51 months [10]. Trastour et al. [5] described a case of rectal recurrence 20 years after the resection of a stage III papillary clear cell OC. IHC study confirmed CK7 positivity and CK20 negativity, with a positive CA-125. The patient received a total proctectomy with protective colostomy. Two years later, abdominal MRI showed a hepatic metastasis. The histopathological exam pointed to a gynecological origin (CK7 +, CK 20 -, CA-125 +). Zighelboim et al. [11] observed the development of a sigmoid tumor six months after left adnexectomy of an ovarian carcinoma with low malignant potential, and no further treatment was recommended. The histology of the sigmoid tumor was suggestive of an ovarian origin; however, it was a high-grade papillary carcinoma with areas of endometrioid carcinoma, the tumor was immunohistochemically positive for CK7+, CEA+, CA-125+, and negative for transcription termination factor 1 (TTF1), and CK20-.

Other site of metastasis, besides colorectal, is the gastric parenchyma which is an extremely rare location, few cases have been reported, Zhou and Miao [12] discussed a case report of 61-year-old women with no clinical manifestation but a high CA-125 and a history of ovarian adenocarcinoma treated, 12 years ago, by surgery + adjuvant chemotherapy. The diagnosis of metastatic OC was confirmed after the performance of gastrectomy. The immunohistochemical staining was positive for WT1, CA-125, CK7 and negative for caudal-related homeobox transcription factor 2 (CDX2). In our case, the rectal metastasis occurred earlier and discovered at the operation of the primary tumor.

The particular feature of our case is that the patient has, in addition to rectal metastasis, a right supra clavicular adenopathy resulting from a histologically confirmed OC. Only few cases have been described in the literature with this trait. It is observed that lymph node metastases are present in 70 % of the patients, however supra clavicular metastases are reported in only 4 % of them, this is partly explained by the connection of the two supra and sub diaphragmatic stages and the flow of lymphatic fluid through the lymph nodes until the supra-clavicular nodes [13].

In OC, the rate of complications by fistula is rare, estimated between 0.8 % and 8.2 % [14]. It is affected by many factors including the development of ascites greater than 500 mL [15], hypoalbuminemia less than 3 g/dL [14] or the use of targeted therapy [16]. It has been reported in the literature that a recto-vaginal fistula can appear when introducing an antiangiogenic treatment [17], [18]. Bevacizumab has confirmed its efficacy especially in terms of progression-free survival [19]. However, the use of this molecule seems to enhance certain complications. Chereau et al. [20] discussed the first case of recto-vaginal fistula in a 45-year-old woman treated for OC and who complained of having stools through the vagina after 2 cycles of bevacizumab. Therefore, they recommended some precautions; first, bevacizumab should be introduced in the second cycle of chemotherapy and secondly, an ileostomy may be suggested for selected individuals. Presumably, this is the first study suggesting that recto-vaginal fistula may be developed spontaneously regardless of any established risk factors.

## 4 Conclusion

When an OC is associated with colorectal cancer, pathologic diagnosis is key to identify which one is the primary tumor, using immunohistochemical staining with cytokeratin 7 and 20. The IHC based finding can provide an optimal therapeutic approach. It is essential that clinicians bear in mind that a proper peri-operative assessment of the gastrointestinal tract should be performed for ovarian tumors to exclude synchronous lesions. The occurrence of a spontaneous rectovaginal fistula is an uncommon complication that should be considered while treating advanced ovarian neoplasia; this situation highlights the importance of periodic follow-up of patients with unusual metastatic sites.

## Consent to participate

Written informed consent was obtained from the patient for publication of this case report and accompanying images. A copy of the written consent is available for review by the Editor-in-Chief of this journal on request.

## Provenance and peer review

Not commissioned, externally peer-reviewed.

## Ethical approval

Ethical approval has been exempted by our institution.

## Funding

None.

## Guarantor

Kharmach Ikram.

## Research registration number

Not applicable.

## CRediT authorship contribution statement

Kharmach Ikram: Writing, review and editing of the manuscript.

Malki Samia: interpretation of histological data.

Al Jarroudi Ouissam: supervised the writing of manuscript.

El Harroudi Tijani: contributor.

Serji Badr: supervision and surgeon of the patient.

Afqir Said: supervision and data validation.

## Declaration of competing interest

The authors have no conflict of interest to declare.
